# Mesothelioma Due to Workplace Exposure: A Comprehensive Bibliometric Analysis of Current Situation and Future Trends

**DOI:** 10.3390/ijerph20042833

**Published:** 2023-02-06

**Authors:** Hanpeng Lai, Chenglei Hu, Man Qu, Xing Liu, Yu Xue, Ping Xu, Dongdong Hao

**Affiliations:** 1 Department of Occupational and Environmental Health, School of Public Health, Yangzhou University, Yangzhou 225009, China; 2 Department of Radiology and Functional Examination, Nanjing Prevention and Treatment Center for Occupational Diseases, Nanjing 210018, China; 3 Lanzhou 7th Rest Center for Retired Cadre, Gansu Military Region, Lanzhou 730000, China

**Keywords:** mesothelioma, workplace exposure, bibliometric analysis, web of Science, VOSviewer

## Abstract

**Background:** This article provides an overview of the current status and research progress of mesothelioma. **Methods:** A total of 2638 documents published from 1 January 2004 to 30 November 2022 were retrieved from the Web of Science Core Collection and analyzed via Microsoft Office Excel 2019, VOSviewer 1.6.18, and Tableau 2022.2. **Results:** There was an obvious increase in the number of publications regarding mesothelioma in the last 18 years, with the United States dominating the research field with 715 publications and 23,882 citations, while the University of Turin contributed the most (118). *Occupational & Environmental Medicine* was the most popular journal (80), with Corrado Magnani being the most prolific author (52) and Michele Carbone obtaining the most citations (4472). “Oncology” and “Health Science of Environment & Occupation” were the two main subjects, while the keywords “asbestos”, “lung cancer”, “gene expression”, “apoptosis”, “survival”, and “cisplatin” were the most popular. **Conclusions:** The containment of mesothelioma calls for more participation from low- and middle-income countries, and further attention needs to be paid to clinical research.

## 1. Introduction

Mesothelioma is a type of carcinoma originating from thin-layer tissues that cover the internal organ surface [1]. As a rare and highly aggressive tumor, it mainly affects the pleura, occasionally the peritoneum, pericardium, and tunica vaginalis [2]. More than 3000 new mesothelioma cases are diagnosed annually in the United States, with an incidence of nearly 30 cases per million in Australia and Great Britain [3]. Mortality rates of mesothelioma have been estimated to increase yearly by 5% to 10% in most industrialized countries until the end of 2020 [4], resulting in an average of 17 potential years of life lost [5]. However, the hidden burden of disease in underdeveloped countries will lead to an underestimation of global incidence and mortality [6]. In China, about 2000 cases and 1600 deaths arise each year [7]. Mesothelioma patients are often diagnosed at an advanced stage, with a median survival of merely 12 months, largely attributed to the insidious onset and initial ambiguous symptoms [8]. Chemotherapy is the standard treatment for mesothelioma, despite its limited effects on improving patient prognosis, and the novel targeted therapy is challenging due to a lack of suitable therapeutic targets [9].

Exposure to asbestos in worksites has been well documented as the primary risk factor for mesothelioma [10]. Inhalation of asbestos fibers can trigger interconnected pathways of oxidative stress, chronic inflammation, tumor signaling activation, and aberrant apoptosis, which induce tumorigenesis [11]. As a recent investigation has found, 80% of patients with malignant pleural mesothelioma reported a history of direct or indirect asbestos exposure [12]. It has been speculated that approximately 125 million laborers globally have a history of asbestos exposure [13]. Despite the ban on the use of asbestos in over 66 countries and territories, the global incidence of malignant mesothelioma will continue to increase due to the long incubation period of 20 to 40 years from first exposure to tumor onset [14]. Additionally, the unregulated use of asbestos in countries such as India, Brazil, and Russia will increase the severity and difficulty of mesothelioma surveillance [15]. Male workers, particularly those more than 65 years old, are more predisposed to mesothelioma than females [14].

Bibliometrics is a review method that evaluates the current status and future progress of a specific research field by quantitatively evaluating the publication characteristics of literature, such as co-citation and co-occurrence relationships, with the help of statistical techniques [16,17,18,19]. Several bibliometric studies have been performed regarding the health effects of asbestos exposure [20,21,22]. However, no bibliometric analyses have focused exclusively on mesothelioma until now, although mesothelioma research has made great progress over the past ten years. To fill this gap, this study aimed to review the information regarding historical trends, inter-agency collaborations, author and article citations, as well as keyword co-occurrences in mesothelioma research due to workplace exposure and perspectives of future progress trends.

## 2. Materials and Methods

### 2.1. Data Collection

**Figure 1:**
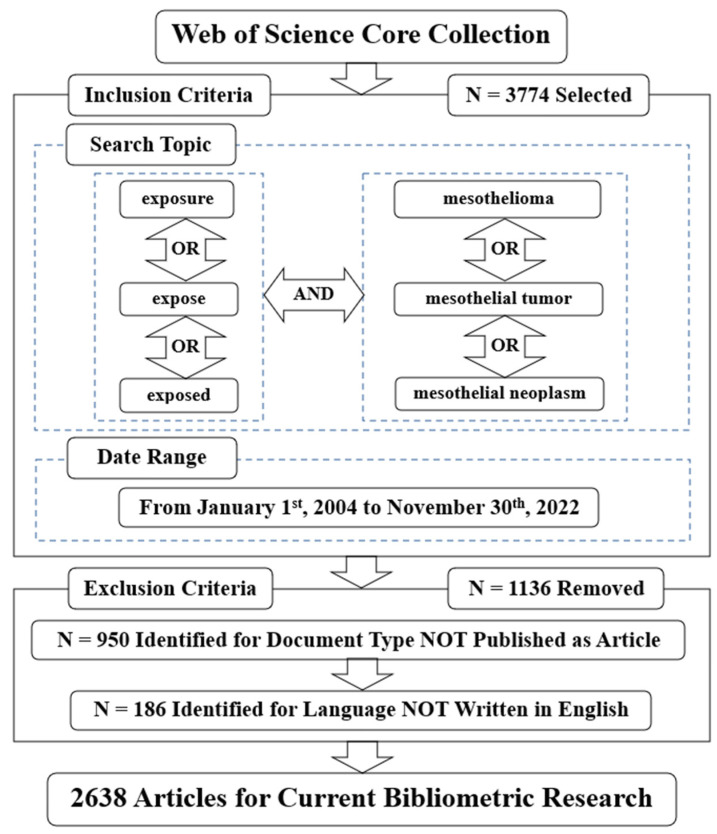
The search strategy.

The data utilized for this study were sourced from the Web of Science Core Collection database in December 2022, since it is the most trustworthy publisher-independent citation database throughout the world, being traced back to the 1990s or earlier and covering more than 13,000 highly influencing authoritative academic journals across multiple disciplines while containing nearly all of the leading global publications on workplace safety and industrial hygiene [23]. In contrast, other types of databases, such as Scopus, only provide published literature in recent years, which have been found not to fully meet the strict screening criteria, and hence the impact factors are generally lower [17,18]. The search strategy is displayed in Figure 1, and the search terms were TS = (“exposure” OR “expose” OR “exposed”) AND TS = (“mesothelioma” OR “mesothelial tumor” OR “mesothelial neoplasm”). The data range was limited “from 1 January 2004 to 30 November 2022.” The language was refined as “English”, and the document type was refined as “Article” because they usually involved more comprehensive information and occupied higher quality rankings [24]. The Web of Science Document Information Management Online System was applied to output the literature information data in the form of “Full Records and Cited References” [25]. All query tasks were accomplished within the same day to prevent potential bias caused by the dynamic database update, and 2638 articles were retrieved.

### 2.2. Data Analysis

The results of the bibliometric analysis were tabulated and visualized. Microsoft Office Excel 2019 was adopted for trend analysis of the number of annual publications from 2004 through 2021 (issuance status of 2022 could merely be traced till the end of November) and to summarize the institutions, funding agencies, journals, and authors with the top 15 publications as well as publications with the top 15 citations. Through VOSviewer 1.6.18, the results were visualized as network or overlay maps [26], with different colors used to indicate various clusters of countries, institutions, authors, publications, or keywords, while linking lines indicated co-authorship, co-occurrence, or co-citation between them (Supplementary Materials). The node size measured the significant degree of each element, and the line thickness measured the strength of collaboration. When setting the overlap visualization, different colors indicated the average published year of each element, and elements in blue appeared the earliest and in red appeared the latest. The worldwide distribution of publications and citations was observed via Tableau 2022.2, which was also used to map word clouds of related research areas. To extract the required results from the collected data, the current bibliometric analysis was again ensured to cover five main stages: defining search criteria, collecting suitable literature, describing volume trends, integrating and transforming data, and visualizing network correlation [18].

## 3. Results

### 3.1. Annual Publication Trends

**Figure 2:**
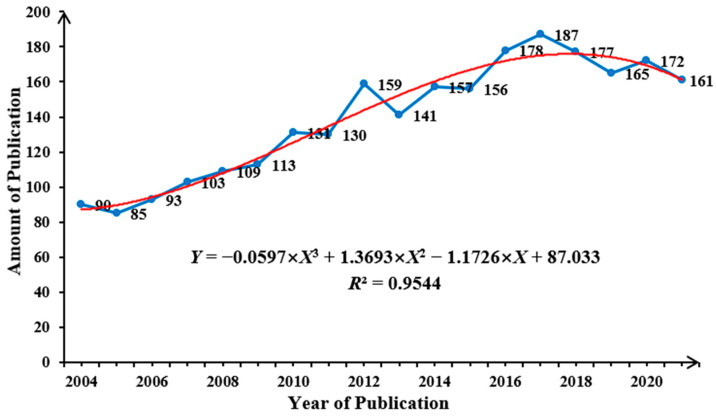
The annual trend in publications regarding mesothelioma due to workplace exposure.

The annual trend of publications regarding mesothelioma due to workplace exposure from 2004 to 2021 is illustrated in Figure 2. Despite some fluctuations, there was an increment trend, in the long run, approximated by a cubic polynomial growth curve corresponding to the equation of *Y* = −0.0597 × *X*^3^ + 1.3693 × *X*^2^ − 1.1726 × *X* + 87.033, with *R*^2^ = 0.9544. The article output was small and averaged less than 100 before 2007, especially in 2005, when only 85 articles were published. Since 2008, the yield has significantly increased, and 2012 saw a historic breakthrough of approaching 160 articles. Annual production steadily surpassed 175 in 2016, peaked at 187 in 2017, and slightly decreased to 161 in 2021. The total annual average number was 139.28, while the average number of publications in the last five years was 172.4. This increase indicates that interest in the issue of exposure-related mesothelioma has gradually increased. It is worth noting that by 2022, 131 articles had already been published by the end of November.

### 3.2. Country, Institution, and Funding Agency Contributions

**Figure 3:**
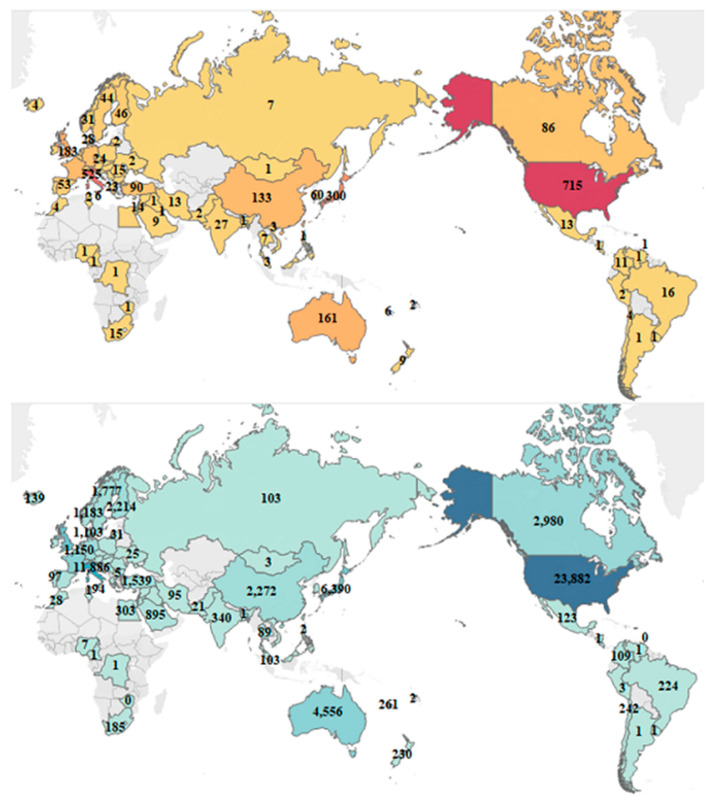
The worldwide distribution of publications (**upper**) and citations (**lower**) regarding mesothelioma due to workplace exposure.

The number of publications and citations concerning mesothelioma due to workplace exposure in each country is presented in Figure 3. Traditional industrial powerhouses, such as the United States, Italy, Japan, the United Kingdom, Australia, and France, as well as newly industrialized countries such as China and Turkey, were actively engaged in mesothelioma research. The United States produced the most articles (715 articles; 27.10%), followed by Italy (525; 19.90%) and Japan (300; 11.37%), with the United States ranked first for citations (23,882 in total, with an average of 33.40).

The top 15 institutions with the most mesothelioma publications are listed in Table 1, with seven institutions located in Italy and the rest situated in Australia, France, and the United States. The University of Turin (118 articles, accounting for 4.47%) held the biggest share of publications, followed by the University of Western Australia (85 for 3.22%) and the Amedeo Avogadro University of Eastern Piedmont (84 for 3.18%). The most frequent citation was also observed at the University of Turin (2938 in total, with an average of 24.90).

In addition, the top 15 funding agencies supporting the publications are shown in Table 2, with six funding agencies attributed to the United States and the others belonging to Japan, Australia, and European countries. The U.S. Department of Health and Human Services funded the largest proportion of published articles (235, making up 8.91%) and obtained the highest number of citations (11,150 in total, with an average of 47.45). The National Institutes of Health and the National Cancer Institute, both in the United States, sponsored the second and third most published articles (209 and 136 papers, making up 7.92% and 5.16% of the total, respectively).

### 3.3. Co-Authorship across Countries and Institutions

**Figure 4:**
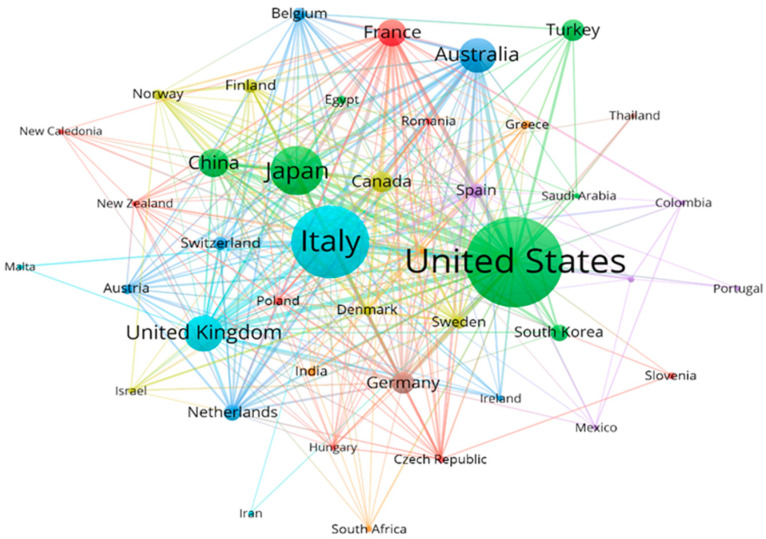
Network visualization of co-authorship across countries. Only countries with at least five documents and 50 citations were selected, and 42 met the thresholds. The size of the nodes indicated the degree of collaboration, and the width of linking lines indicated the collaboration strength. Countries of the same color indicated closer co-authorships.

The network visualization of co-authorship across countries is depicted in Figure 4. The United States occupied the vast majority of partnerships with a total link strength of 368 and maintained a strong cooperative relationship with Italy, Australia, France, and the United Kingdom, all of which showed a total link strength greater than 150 with other countries. Except for the industrialized countries mentioned above, China, as a rising power, also revealed extensive participation in international collaboration with a total link strength of 114.

**Figure 5:**
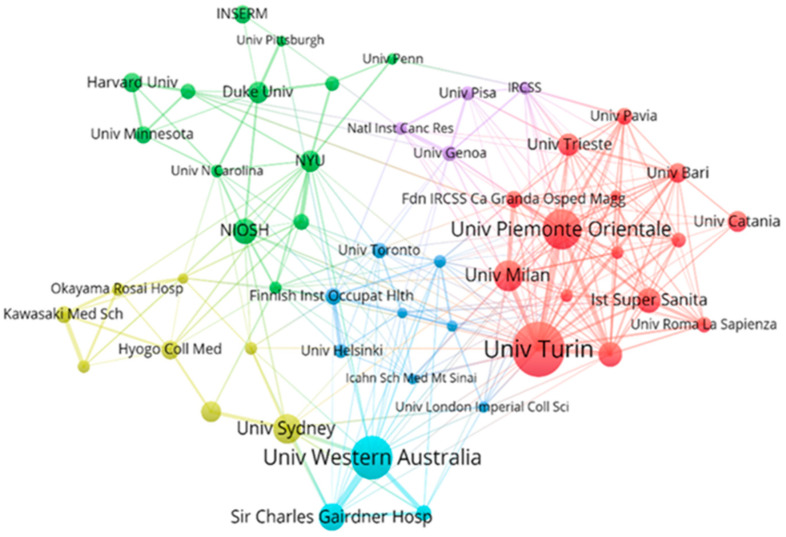
Network visualization of co-authorship across institutions. Only institutions with at least 15 documents and 50 citations were selected, and 51 met the thresholds. The size of the nodes indicated the degree of collaboration, and the width of linking lines indicated the collaboration strength. Institutions of the same color indicated closer co-authorships.

The co-authorship network among institutions is provided in Figure 5. The top two institutions with the most extensive cross-agency collaboration were both in Italy, that is, the University of Turin and the Amedeo Avogadro University of Eastern Piedmont, with total link strengths of 78 and 63, respectively, followed by the University of Western Australia and Sir Charles Gairdner Hospital, both established in Australia and fully dedicated to inter-agency cooperation on related issues with total link strengths of 56 and 43, respectively.

### 3.4. Journal and Author

The top 15 journals with the highest publication regarding mesothelioma due to workplace exposure are listed in Table 3, which holds for nearly one-quarter of the total publication amount. Some journals were deleted due to publisher acquisition, name change, or delisting from Journal Citation Reports. Nearly half of the listed journals were based in the United States. *Occupational & Environmental Medicine* (80 articles making up 3.03%) appeared to be the most author-friendly journal, also taking the first position of citations (2925 in total), followed by the *American Journal of Industrial Medicine* (74 for 2.81%). *Lung Cancer* (51 for 1.93%) and the *International Journal of Environmental Research & Public Health* (49 for 1.86%) published the third and fourth most research articles. The high-impact journals concerned the diagnosis and therapy of oncological diseases. The *Journal of Thoracic Oncology* earned the highest score in 2021 (IF of 20.121), while *Cancer Research* and the *British Journal of Cancer* were also found with good citations (IF of 13.312 and 9.075, respectively).

The top 15 authors with the most publications are presented in Table 4, with almost half of the authors conducting research activities in Italy, followed by Australia and the United States. Corrado Magnani made the most contributions (52 articles for 1.97%) at the University of Eastern Piedmont Amedeo Avogadro, followed by Dario Mirabelli (51 for 1.93%) at the University of Turin. Regarding citations, Michele Carbone and Harvey I. Pass held the position of upper rank two (4472 and 4276 in total, respectively), taking office in the University of Hawaii System and New York University, respectively. All the above authors also had H-indices greater than 20.

### 3.5. Highly Cited Articles

The top 15 published articles with the most citations are shown in Table 5. The article “Carbon nanotubes introduced into the abdominal cavity of mice show asbestos-like pathogenicity in a pilot study” by Craig A. Poland et al., published in *Nature Nanotechnology* in 2008, acquired the most citations of 1875 [27]. The article “Germline BAP1 mutations predispose to malignant mesothelioma” by Joseph R. Testa et al. in *Nature Genetics* in 2011 only obtained 674 citations [28], and “Induction of mesothelioma in p53+/− mouse by intraperitoneal application of multi-wall carbon nanotube” by Joseph R. Testa et al. in the *Journal of Toxicological Sciences* in 2008, which had 591 citations [29].

### 3.6. Co-Citations across Cited Authors and Cited References

**Figure 6:**
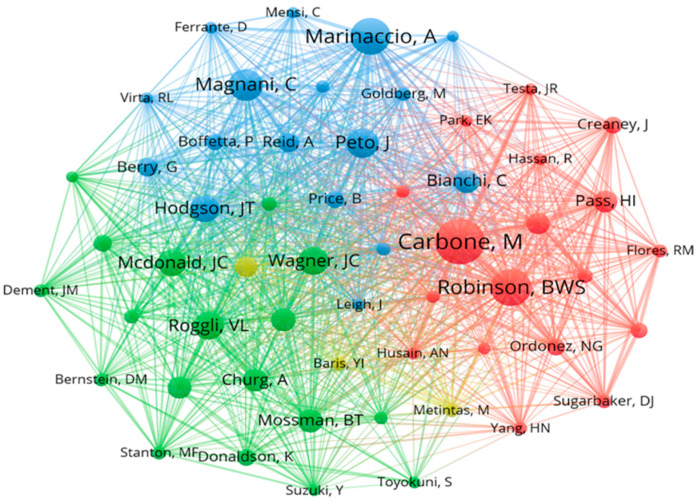
Network visualization of co-citation across cited authors. Only cited authors with at least 200 citations were selected, and 58 cited authors met the thresholds. The name of the institutes was deleted. The width of the lines linking the nodes indicated the closeness of co-citation. Authors of the same color indicated closer co-citations.

The co-citation analysis network among authors is provided in Figure 6. The name of the institution, e.g., the International Agency for Research on Cancer, was deleted. Michele Carbone (hired by the University of Hawaii System) proved to be the most co-cited author (total link strength of 404.85), followed by Bruce W. S. Robinson (University of Western Australia; 370.89) and Alessandro Marinaccio (Climate & Sustainability Foundation; 321.28).

**Figure 7:**
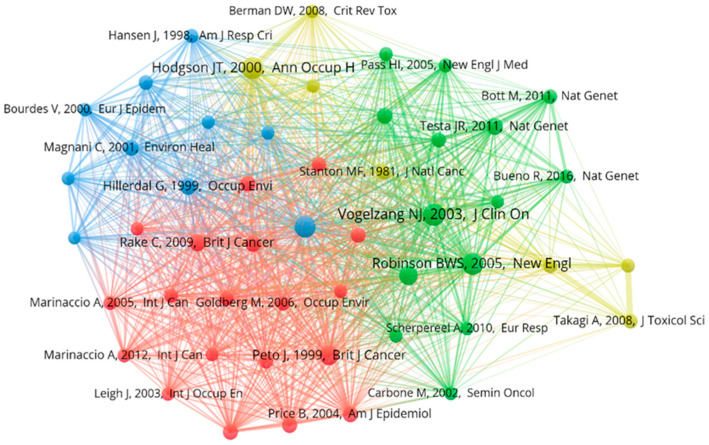
Network visualization of co-citation across cited references. Only cited references [27,28,29,30,31,32,33,34,35,36,37,38,39,40,41] with at least 50 citations were selected, and 51 cited references met the thresholds. The width of the lines linking the nodes indicated the closeness of co-citation. Cited references of the same color indicate closer co-citations.

The co-citation analysis network across cited references is presented in Figure 7. The cited reference “Quantitative risks of mesothelioma and lung cancer concerning asbestos exposure” by John T. Hodgson in *Annals of Occupational Hygiene* in 2000 (now renamed *Annals of Work Exposures and Health*) had the strongest co-citation relationship (total link strength of 165), ref. [42] followed by “Diffuse pleural mesothelioma and asbestos exposure in the North Western Cape Province” by Johanna C. Wagner in the *British Journal of Industrial Medicine* in 1960 (renamed *Occupational & Environmental Medicine;* 152) [43]. 

### 3.7. Research Area

A total of 91 research areas were involved, varying from biology, clinical medicine, public health, and engineering to environmental science. The subjects “Public Environmental Occupational Health” and “Oncology” had the most articles (657 and 640, respectively), greatly ahead of “Toxicology” (276), “Respiratory System” (259), “Environmental Sciences Ecology” (233), and “Pathology” (161).

### 3.8. Clustering Analysis and Time Distribution across Keywords

**Figure 8:**
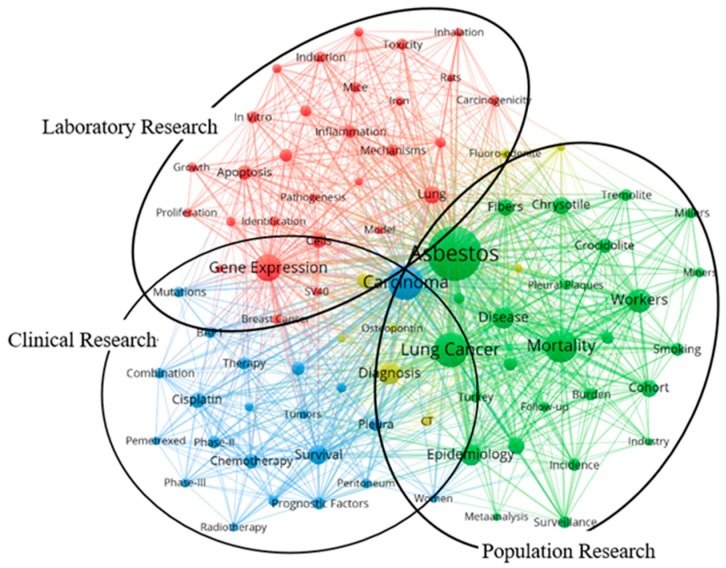
Network visualization of co-occurrence across keywords. Only keywords with at least 25 occurrences were selected, and 115 met the thresholds. Duplicate keywords were merged, and irrelevant keywords were deleted. There were three clusters: laboratory research, clinical research, and population research. The size of the nodes indicated the frequency of occurrence.

The network visualization of keyword co-occurrence is displayed in Figure 8. Duplicate keywords, e.g., “lung cancer” and “lung-cancer”, were merged, whereas irrelevant keywords, e.g., “management” were deleted. The keywords selected in the visualized analysis were finally divided into three clusters: laboratory research, clinical research, and population research. For laboratory research, the main keywords were listed as “gene expression” (293 times of occurrence), “lung” (114), “cells” (88), “apoptosis” (85), “inflammation” (63), “activation” (62), “in vitro” (56), and “mechanisms” (55). For population research, the primary keywords emerged as “asbestos” (1127), “lung cancer” (457), “mortality” (400), “workers” (218), “disease” (174), “epidemiology” (170), “chrysotile” (121), and “cohort” (112). For clinical research, keywords in the forefront were displayed as follows: “carcinoma” (478), “survival” (148), “cisplatin” (96), “chemotherapy” (85), “immunohistochemistry” (69), “pleura” (67), “prognostic factors” (63), and “Phase II” (62).

**Figure 9:**
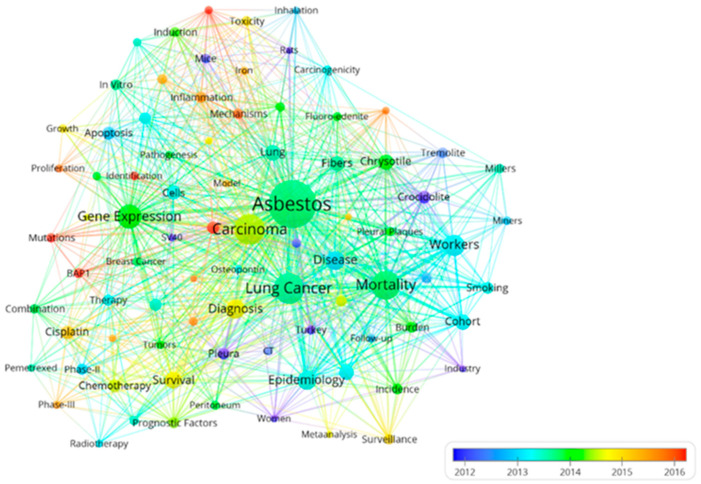
Overlay visualization of time distribution across keywords Only keywords with at least 25 occurrences were selected, and 115 met the threshold. Duplicate keywords were merged, and irrelevant keywords were deleted. The color indicates the average published year of keywords, with keywords in blue appearing as the earliest and in red appearing as the latest.

The overlay visualization of time distribution across keywords is depicted in Figure 9. Keywords of population research appeared in the earliest period, with an average published year of 2013.25, followed by laboratory research (2014.07), and keywords of clinical research came up in the latest period (2014.33). During the earlier stage, “industry” and “Turkey” were the first to attract scholars’ attention (average published years of 2010.08 and 2011.76), while “erionite”, “tremolite”, and “crocidolite” seemed to be the most prevalent topics (44, 58, and 86 times of occurrence). During the latter stage, “BAP1”, “mutations”, and “prognosis” came to public notice in later years (average published years of 2018.25, 2016.60, and 2015.72), while “chemotherapy”, “cisplatin”, and “survival” were hot topics (85, 96, and 148 times of occurrence).

## 4. Discussion

Bibliometric analysis is an excellent approach for revealing the current status of relevant issues in specific research areas and predicting future development trends [44]. Compared with systematic reviews and meta-analyses, although bibliometrics is not easy to extract novel ideas via investigating previous findings, it provides quantitative criteria with minimal potential bias, contributing to time-saving, low-cost, and repeatable evaluations for interdisciplinary readers. Depending on the Java environment, VOSviewer has been commonly accepted by researchers as efficient software for constructing and visualizing bibliometric networks with functional modules of multi-dimension, time-sharing, and dynamic citation [26]. Articles analyzed in this study were exclusively selected via the Web of Science Core Collection database because it contains the most comprehensive journal types and covers almost all traditional and emerging disciplines worldwide [23].

Mesothelioma is a fatal invasive carcinoma prevalent in occupationally exposed workers, most of which arise in the pleura, and there is currently no effective treatment available [45]. Asbestos has been identified as the principal cause, whose carcinogenic process begins with infiltration in normal mesothelial cells, inducing the accumulation of macrophages as well as the sustained release of inflammatory factors, and finally resulting in the malignant transformation into mesothelioma cells [46]. As estimated by the World Health Organization, nearly 43,000 mesothelioma sufferers die annually [5]. Given the severe health effects caused by mesothelioma among industrial workers, it is necessary to summarize the results of previous research to integrate valuable information so that more scientific interventions may be put into action. This study intended to evaluate findings regarding mesothelioma caused by exposure to hazardous substances across countries, institutions, funding agencies, journals, authors, and published articles, while providing an overview of research hotspots over the years for perspectives on future trends.

The annual publication number fluctuated, with little change in the annual output before 2009, and a record low of 85 in 2005. Subsequently, the number of publications started to increase, especially in 2017, which witnessed the highest production in history. Meanwhile, the years after 2017 also accounted for more than half of the total publications, corresponding to a relatively large average amount in the last five years, which went beyond 170. The above findings suggest that 2017 was the watershed year for mesothelioma research. Breakthrough progress in the pathology and genetics research of pleural mesothelioma beginning in 2016 contributed to the explosive growth of findings in overcoming the problems of mesothelioma treatment [47]. In addition, as an investigation in 2018 by the World Health Organization pointed out, although some improvements had occurred since specific interventions were given, mesothelioma remained a major disease burden and the leading cause of mortality on a global scale [48]. The number of published articles in 2022 was 131, merely covering the data of the 11 months; however, it is foreseeable that the annual yield will maintain an upward trend.

Most of the findings originated in several developed countries, whose years of effort have also prevented the onset of mesothelioma in western countries to some extent [49]. The United States was the leading country in the mesothelioma research field, as evidenced by its dominance in publications (715), citations (23,882), and the H-index (74). Among the top 10 contributive countries, only two emerging economies, China, and Turkey, were on the list. While chrysotile has been broadly used by the Chinese textile industry for a few decades, investigations on the association of exposure to chrysotile with mesothelioma incidence in China are still insufficient [50], particularly for female laborers [51]. An epidemiological study revealed that malignant mesothelioma is gradually becoming the major occupational neoplasm associated with asbestos exposure among Chinese workers [7]. In contrast, Turkey earlier identified the adverse effects of mesothelioma induced by fibrous minerals [52], partly attributed to the alarmingly high prevalence in Central Anatolia [53]. Nevertheless, academic institutions in Italy [54] and Australia [55] were more actively engaged in collaboration (nine of the top 15 institutions), where nationwide registries and surveillance systems had been established. The funding agencies in the United States were the most enthusiastic about sponsoring relevant research (six of the top 15 funding agencies), having been committed to improving mesothelioma patient prognosis over the years [56]. The containment of mesothelioma incidence requires more participation from newly industrialized countries.

The top 15 highly yielding journals published 22.53% of the total articles, involving two major challenges to modern medicine, “oncology” and “environmental health”. *Occupational & Environmental Medicine* and the *American Journal of Industrial Medicine* were the most sought-after journals for mesothelioma researchers, with an overwhelming advantage in the publication rankings (both greater than 70). *Occupational & Environmental Medicine* has focused on exposure and health impact assessment of workplace potential hazards, e.g., ambient air pollutants, while the *American Journal of Industrial Medicine* focuses on employee mental health and labor policy reforms. Another bibliometric study of asbestos exposure offered similar results [21]. Six of the 15 listed journals with impact factors greater than five published 173 articles (32.10% of the included publications), whereas two journals with impact factors were below three and only published 50 articles (9.28%). Consequently, research topics on mesothelioma have become the main concern of influential journals.

Italian scholars maintained the highest productivity regarding mesothelioma (seven of the top 15 authors). Corrado Magnani was the most contributive scientist (52 articles) in conquering mesothelioma, with research interests in the health effects of asbestos exposure and the prevalence of mesothelioma. A recent multicenter study by Corrado Magnani found an obvious increase in pleural mesothelioma risk in industries with high consumption of asbestos [57]. Among the top 15 prolific authors, two American scientists were far ahead in citations (both over 4000), suggesting that academic groups in the United States consider research quality equally important to the number of publications. Michele Carbone took the leading position in both cited and co-cited authors and has been committed to revealing the underlying causes of asbestos carcinogenic differences in various exposed populations. As Michele Carbone discovered, synergy SV40 with asbestos in a reduced dose would lead to the incidence of mesothelioma [58]. In general, an in-depth exploration of mesothelioma requires more collaboration from scholars in other countries.

The 15 most frequently cited articles consisted of eight mechanism explorations, six population surveys, and only one clinical trial, indicating a lack of high-level clinical findings for mesothelioma treatment. The article “Carbon nanotubes introduced into the abdominal cavity of mice showed asbestos-like pathogenicity in a pilot study” was most cited and revealed the potential adverse effects of inducing mesothelioma due to carbon nanotube exposure [27]. Another paper, “Germline BAP1 mutations predispose to malignant mesothelioma”, confirmed the existence of a BAP1-associated cancer syndrome characterized by mesothelioma [28], providing foundations for future studies evidenced by its top-ranking citations and co-citations [59]. Highly cited articles were mainly distributed in journals with higher impact factors; for example, the two articles in the leading position of citations were published by *Nature Nanotechnology* (impact factor of 40.523) and *Nature Genetics* (41.307). Nevertheless, there are still exceptions; for example, the third most cited publication, “Induction of mesothelioma in p53+/− mouse by intraperitoneal application of multi-wall carbon nanotube”, was published in the *Journal of Toxicological Sciences* (1.792). Given previous findings, the accumulation of cutting-edge results in the future will contribute to the remarkable development of mesothelioma treatment.

### 4.1. Frontiers and Perspectives

Mesothelioma research has chiefly focused on two subjects, “Health Science of Environment & Occupation” and “Oncology”, while the disciplines of “Toxicology”, “Respiratory Science”, and “Ecology & Environmental Science” also brought together plenty of relative studies. In the current study, keywords of high frequency associated with mesothelioma were considered predictive factors of research frontiers.

For population research, “asbestos”, “lung cancer”, and “mortality” appeared as high-frequency words, consistent with the contemporary knowledge on mesothelioma prevalence. Asbestos has been identified by the International Agency for Research on Cancer as an occupational hazard for mesothelioma [10], regardless of its mortality rate being lower than asbestos-related lung cancer [60]. Nevertheless, after 20 years of exposure cessation, there was a reduced risk of lung cancer, while the risk of mesothelioma showed no change [61]. Given these severe health effects, 66 countries and territories have banned the application of asbestos [62]. The mesothelioma burden varied globally across countries and was predominantly reflected in situations among high-income nations [63]. Hence, the stringent containment of mesothelioma requires the cooperation of more low- and middle-income countries with sufficient and detailed data.

For laboratory research, “gene expression”, “apoptosis”, and “inflammation” attracted the most public attention since they are considered driving factors for tumorigenesis [64]. BAP1 is the most commonly inactivated gene in mesothelioma, the deficiency of which triggers cancerous transformation through disruption of DNA repair, transcription regulation, and apoptosis [28]. In addition to BAP1, recent studies have confirmed an increasing number of candidate genes that predispose patients to developing mesothelioma [65]. Biomarkers of systemic inflammation have been verified to predict the prognosis of malignant mesothelioma [66], and poor patient outcomes have been attributed to suppression of the specific immune system as well as activation of its innate counterpart [67], providing new ideas for improving the life quality of patients.

For clinical research, “survival”, “cisplatin”, and “chemotherapy” became academic focuses as a result of the urgent need for mesothelioma treatment. Mesothelioma patients have a median survival rate of only 12 months, and the lack of appropriate biomarkers has become a bottleneck for subsequent therapy [68]. Chemotherapy is the established standard for mesothelioma treatment, mainly combined with cisplatin and pemetrexed [69]. Besides, increasing knowledge about the molecular characteristics of mesothelioma has resulted in the discovery of novel potential targets for systemic therapy. However, there is still a long way to go from the discovery of molecular targets to the development of potential wonder drugs [70]. Apart from traditional therapy, immunotherapy has recently been demonstrated as a novel and promising treatment for mesothelioma that harnesses the power of the human immune system [71].

As the time distribution suggested, keywords of population studies appeared in the earliest stage, followed by laboratory studies and clinical studies, which was consistent with the previous trends in mesothelioma research. Early scholars engaged in mesothelioma research mainly concentrated on population studies. With the accumulated epidemiological evidence and widely recognized dose-response relationship, many researchers have turned to mechanistic exploration based on in vivo and in vitro experiments. Emerging laboratory findings have provided theoretical support for subsequent clinical research, greatly improving the quality of life of mesothelioma patients. However, there is still a lack of influential clinical research; hence, there is a need for more clinical studies.

### 4.2. Advantages and Limitations

To the best of our understanding, this is the first bibliometric analysis of mesothelioma over the past decade. A similar bibliometric study was published in 2010, merely reviewing previous research progress by the end of 2006 [22]. Our current findings are more objective and comprehensive since they were obtained from the Web of Science Core Collection database, the world’s most trusted citation database, with more than 13,000 highly influential academic journals. All the retrieval tasks were accomplished on 9 December 2022, to avoid potential bias caused by the dynamic update of the database.

However, this study has some limitations. The literature included in our analysis was articles written in English retrieved from the Web of Science, so papers written in languages other than English, editorials, letters, and meeting abstracts were excluded. Also, other medical literature databases, such as PubMed, Scopus, and Google Scholar, that provided publications available before 2004, were not searched, possibly resulting in a data source reduction and cognitive bias of the current situation on mesothelioma.

## 5. Conclusions

There has been a marked increase in the number of publications regarding mesothelioma in the last 18 years, especially from 2008. The United States was the dominant country in the research field, with the most publications and citations. The University of Turin was the most active institution, and the U.S. Department of Health and Human Services sponsored the largest proportion of articles. *Occupational & Environmental Medicine* was the most popular journal for scholars, with Corrado Magnani being the most prolific author and Michele Carbone obtaining the most citations. The mesothelioma research mainly involved two disciplines, “Oncology” and “Health Science of Environment & Occupation”, while the keywords “asbestos”, “lung cancer”, “gene expression”, “apoptosis”, “survival”, and “cisplatin” were considered hot topics. Mesothelioma research requires more participation from low- and middle-income countries, as well as more clinical research.

## Figures and Tables

**Table 1:** Top 15 institutions with the highest publications regarding mesothelioma due to workplace exposure.

Institution	Document (%)	Citation	^#^ Country	H-Index
University of Turin	118 (4.47)	2938	Italy	34
University of Western Australia	85 (3.22)	2407	Australia	30
Amedeo Avogadro University of Eastern Piedmont	84 (3.18)	2347	Italy	31
* National Institute of Health and Medical Research	71 (2.69)	2812	France	26
Udice French Research Universities	66 (2.50)	2260	France	23
Harvard University	59 (2.24)	2104	USA	26
Centers for Disease Control and Prevention	55 (2.08)	2442	USA	23
University of Sydney	52 (1.97)	1655	Australia	23
* Piedmont Reference Center for Epidemiology and Cancer Prevention	52 (1.97)	1444	Italy	25
University of Milan	52 (1.97)	1185	Italy	19
University of Genoa	48 (1.82)	1778	Italy	25
* IRCCS Ca Granda Foundation-Maggiore Policlinico Hospital	47 (1.78)	867	Italy	15
New York University	46 (1.74)	4318	USA	31
National Institute for Occupational Safety and Health	46 (1.74)	2442	USA	20
* National Institute of Health	46 (1.74)	855	Italy	18

* The original name of institutions was interpreted into English. ^#^ The USA refers to the United States of America, and the UK refers to the United Kingdom.

**Table 2:** Top 15 funding agencies with the highest publications regarding mesothelioma due to workplace exposure.

Institution	Document (%)	Citation	^#^ Country	H-Index
Department of Health and Human Services	235 (8.91)	11,150	USA	57
National Institutes of Health	209 (7.92)	10,319	USA	53
National Cancer Institute	136 (5.16)	7647	USA	48
Ministry of Education, Culture, Sports, Science, and Technology	109 (4.13)	2741	Japan	29
Society for the Promotion of Science	78 (2.96)	1889	Japan	23
National Institute of Environmental Health Sciences	75 (2.84)	3013	USA	33
European Commission	63 (2.39)	3676	Belgium	26
Grants-in-Aid for Scientific Research	60 (2.27)	1268	Japan	19
* AIRC-Foundation for Cancer Research	52 (1.97)	2060	Italy	26
Ministry of Health, Labour and Welfare	47 (1.78)	2226	Japan	22
Centers for Disease Control and Prevention	37 (1.40)	1192	USA	16
National Health and Medical Research Council	36 (1.36)	830	Australia	18
National Institute for Occupational Safety and Health	30 (1.14)	1077	USA	14
UK Research and Innovation	25 (0.95)	2929	UK	16
Medical Research Council	22 (0.83)	2895	UK	15

* The original name of institutions was interpreted into English. ^#^ The USA refers to the United States of America, and the UK refers to the United Kingdom.

**Table 3:** Top 15 journals with the highest publications regarding mesothelioma due to workplace exposure.

Journal	Document (%)	Citation	* IF in 2021	^#^ Country
Occupational and Environmental Medicine	80 (3.03)	2925	4.948	UK
American Journal of Industrial Medicine	74 (2.81)	1269	3.079	USA
Lung Cancer	51 (1.93)	1264	6.081	Ireland
International Journal of Environmental Research and Public Health	49 (1.86)	263	4.614	Switzerland
Journal of Thoracic Oncology	36 (1.36)	1460	20.121	USA
Inhalation Toxicology	32 (1.21)	489	3.011	UK
Journal of Occupational and Environmental Medicine	31 (1.18)	430	2.306	USA
Regulatory Toxicology and Pharmacology	28 (1.06)	492	3.598	USA
PLOS One	27 (1.02)	837	3.752	USA
BMC Cancer	26 (0.99)	468	4.638	UK
Cancer Research	22 (0.83)	1638	13.312	USA
Cancers	22 (0.83)	118	6.575	Switzerland
British Journal of Cancer	21 (0.80)	1252	9.075	UK
Cancer Science	21 (0.80)	534	6.518	USA
International Archives of Occupational and Environmental Health	19 (0.72)	236	2.851	Germany

* IF refers to the impact factor. ^#^ The USA refers to the United States of America, and the UK refers to the United Kingdom. The names of certain journals were deleted due to publisher acquisition, name change, or delisting from Journal Citation Reports.

**Table 4:** Top 15 authors with the highest publications regarding mesothelioma due to workplace exposure.

Author	Document (%)	Citation	Institution	^#^ Country	H-Index
Corrado Magnani	52 (1.97)	1431	University of Eastern Piedmont Amedeo Avogadro	Italy	24
Dario Mirabelli	51 (1.93)	1704	University of Turin	Italy	28
Alessandro Marinaccio	51 (1.93)	1145	* Italian Workers Compensation Authority	Italy	19
Harvey I. Pass	48 (1.82)	4276	New York University	USA	33
Michele Carbone	44 (1.67)	4472	University of Hawaii System	USA	32
Nicholas H. de Klerk	30 (1.14)	1016	University of Western Australia	Australia	20
Enzo Merler	29 (1.10)	1112	University of Padua	Italy	19
Alison Reid	29 (1.10)	765	Curtin University	Australia	15
Pietro Comba	29 (1.10)	499	* National Institute of Health	Italy	14
Carolina Mensi	24 (0.91)	750	* IRCCS Ca Granda Foundation-Maggiore Policlinico Hospital	Italy	14
V.L. Roggli	24 (0.91)	363	Duke University	USA	11
Elisabetta Chellini	23 (0.87)	668	Cancer Prevention and Research Institute	Italy	13
N. Olsen	23 (0.87)	614	University of Western Australia	Australia	13
Takumi Kishimoto	23 (0.87)	481	Okayama Rosai Hospital	Japan	12
Jean-Claude Pairon	21 (0.80)	635	* Paris-Est Creteil Val-de-Marne University	France	12

* The original name of institutions was interpreted into English. ^#^ The USA refers to the United States of America, and the UK refers to the United Kingdom.

**Table 5:** Top 15 published articles regarding mesothelioma due to workplace exposure, with the highest citation.

Author (Year)	Article	Citation	Journal
CA Poland, et al. (2008)	Carbon Nanotubes Introduced into the Abdominal Cavity of Mice Show Asbestos-like Pathogenicity in a Pilot Study [27]	1875	Nature Nanotechnology
JR Testa, et al. (2011)	Germline BAP1 Mutations Predispose to Malignant Mesothelioma [28]	674	Nature Genetics
A. Takagi, et al. (2008)	Induction of Mesothelioma in p53+/− Mouse by Intraperitoneal Application of Multi-wall Carbon Nanotube [29]	591	Journal of Toxicological Sciences
LB Travis, et al. (2005)	Second Cancers among 40,576 Testicular Cancer Patients: Focus on Long-term Survivors [30]	590	Journal of the National Cancer Institute
G. Zalcman, et al. (2016)	Bevacizumab for Newly Diagnosed Pleural Mesothelioma in the Mesothelioma Avastin Cisplatin Pemetrexed Study (MAPS): A Randomised, Controlled, Open-Label, Phase 3 Trial [31]	525	Lancet
P. Vineis, et al. (2014)	Global Cancer Patterns: Causes and Prevention [32]	367	Lancet
JP Ryman-Rasmussen, et al. (2009)	Inhaled Carbon Nanotubes Reach the Subpleural Tissue in Mice [33]	315	Nature Nanotechnology
HI Pass, et al. (2005)	Asbestos Exposure, Pleural Mesothelioma, and Serum Osteopontin Levels [34]	301	New England Journal of Medicine
JT Hodgson, et al. (2005)	The Expected Burden of Mesothelioma Mortality in Great Britain from 2002 to 2050 [35]	278	British Journal of Cancer
PJ Landrigan, et al. (2004)	Health and Environmental Consequences of the World Trade Center Disaster [36]	260	Environmental Health Perspectives
M. Pacurari, et al. (2008)	Raw Single-wall Carbon Nanotubes Induce Oxidative Stress and Activate MAPKs, AP-1, NF-kappa B, and Akt in Normal and Malignant Human Mesothelial Cells [37]	254	Environmental Health Perspectives
SE Mutsaers, et al. (2004)	The Mesothelial Cell [38]	232	International Journal of Biochemistry & Cell Biology
AH Mokdad, et al. (2017)	Trends and Patterns of Disparities in Cancer Mortality among U.S. Counties, 1980–2014 [39]	225	Journal of the American Medical Association
M. Guled, et al. (2009)	CDKN2A, NF2, and JUN Are Dysregulated among Other Genes by miRNAs in Malignant Mesothelioma: A miRNA Microarray Analysis [40]	216	Genes Chromosomes & Cancer
B. Price et al. (2004)	Mesothelioma Trends in the United States: An Update Based on Surveillance, Epidemiology, and End Results Program Data for 1973 Through 2003 [41]	209	American Journal of Epidemiology

## Data Availability

All data for this study can be found in the WOS (https://www.webofscience.com). The original contributions presented in the study are included in the article. Further inquiries can be directed to the corresponding author.

## Supplementary Materials

The following supporting information can be downloaded at: https://www.mdpi.com/article/10.3390/ijerph20042833/s1, “How to operate the VOSviewer software?”.
