# Supplementary material for: Mesothelioma Due to Workplace Exposure: A Comprehensive Bibliometric Analysis of Current Situation and Future Trends
Source: Int J Environ Res Public Health. 2023 Feb 6;20(4):2833. doi: 10.3390/ijerph20042833 (PMC9956900; doi:10.3390/ijerph20042833)
Supplement: Supplementary file 1 [file ijerph-20-02833-s001.zip › ijerph-2168957-supplementary.pdf]

Supplementary Materials

How to operate the VOSviewer software?

The “create map” dialog should be opened and the radio buttons of “Create a map based on bibliographic data” as well as “Read data from bibliographic database files” could be successively selected. The text (.txt) files downloaded previously from WoS should be imported, and thus the type of analysis, e.g., co-authorship, co-citation, and co-occurrence, could be chosen according to the research purpose.”
